# Supplementary material for: Transcriptomic Insights into Mechanisms of Early Seed Maturation in the Garden Pea (Pisum sativum L.)
Source: Cells. 2020 Mar 23;9(3):779. doi: 10.3390/cells9030779 (PMC7140803; doi:10.3390/cells9030779)

Sprint−2(10) vs Zhongw

Global Summary

%DE = 0.89

genes with fdr < 0.2 = 1854 (930 + / 924 −)

genes with fdr < 0.1 = 1711 (852 + / 859 −)

genes with fdr < 0.05 = 1491 (741 + / 750 −)

genes with fdr < 0.01 = 1232 (618 + / 614 −)

genes in genesets = 0

<FC> = 0

<t−score> = −4.26

<p−value> = 0

<fdr> = 0.11

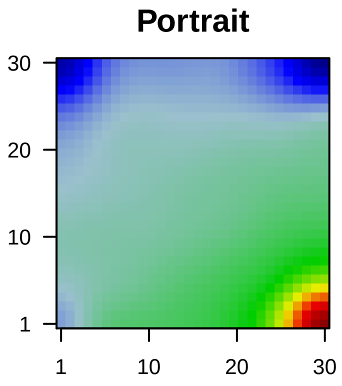

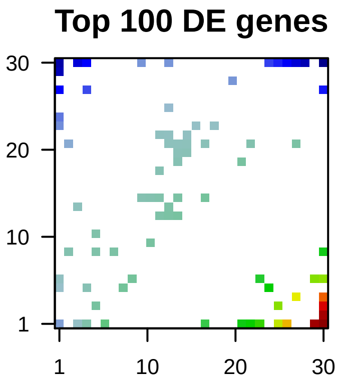

Global Genelist

Rank	ID	log(FC)	p−value	fdr	Metagene	Description
1	comp41855_c	0	4e−12	0.0000000[illegible]	[illegible] x 5	
2	TRINITY_DN4	−4.81	1e−10	0.0000000[illegible]	[illegible] x 1	
3	TRINITY_DN1	−0.1	3e−10	0.0000000[illegible]	[illegible] x 25	
4	TRINITY_DN1	−0.01	5e−10	0.0000000[illegible]	[illegible] x 13	
5	TRINITY_DN2	−0.08	6e−10	0.0000000[illegible]	[illegible] x 3	
6	TRINITY_DN5	−0.01	6e−10	0.0000000[illegible]	[illegible] x 1	
7	TRINITY_DN1	−0.01	9e−10	0.0000001[illegible]	[illegible] x 10	
8	TRINITY_DN1	−0.04	2e−09	0.0000000[illegible]	[illegible] x 27	
9	TRINITY_DN1	−0.09	2e−09	0.0000001[illegible]	[illegible] x 21	
10	TRINITY_DN1	−0.03	3e−09	0.0000000[illegible]	[illegible] x 9	
11	TRINITY_DN1	−0.01	3e−09	0.0000001[illegible]	[illegible] x 13	
12	TRINITY_DN1	−0.03	3e−09	0.0000001[illegible]	[illegible] x 15	
13	TRINITY_DN6	−0.04	3e−09	0.0000001[illegible]	[illegible] x 22	
14	TRINITY_DN1	−0.04	4e−09	0.0000001[illegible]	[illegible] x 21	
15	TRINITY_DN4	−0.03	4e−09	0.0000002[illegible]	[illegible] x 20	
16	TRINITY_DN1	−0.01	6e−09	0.0000002[illegible]	[illegible] x 14	
17	TRINITY_DN2	−0.07	8e−09	0.0000002[illegible]	[illegible] x 22	
18	TRINITY_DN2	0	8e−09	0.0000002[illegible]	[illegible] x 15	
19	TRINITY_DN1	−0.04	9e−09	0.0000002[illegible]	[illegible] x 21	
20	TRINITY_DN2	−0.01	9e−09	0.0000006[illegible]	[illegible] x 15	

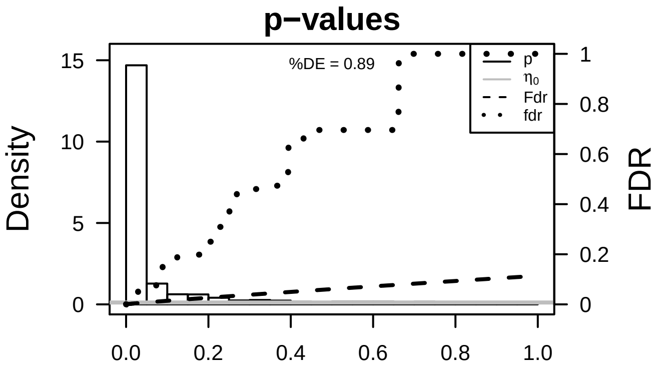

Supplement: Supplementary file 1 [file cells-09-00779-s001.zip › Supplementary materials/FileS4.zip!/FileS3/TFs - Results/Summary Sheets - Differences/Reports/Sprint.2.10..vs.Zhongwan.6.25..pdf]
